# Extracellular Vesicles as a Neprilysin Delivery System Memory Improvement in Alzheimer’s Disease

**DOI:** 10.22037/ijpr.2020.112062.13508

**Published:** 2020

**Authors:** Mehrnaz Izadpanah, Leila Dargahi, Jafar Ai, Afsaneh Asgari Taei, Somayeh Ebrahimi Barough, Seyed Javad Mowla, Gholamreza TavoosiDana, Maryam Farahmandfar

**Affiliations:** a *Department of Tissue Engineering and Applied Cell Sciences, School of Advanced Technologies in Medicine, Tehran University of Medical Sciences, Tehran, Iran. *; b *Neuroscience Research Center, Shahid Beheshti University of Medical Sciences, Tehran, Iran. *; c *Department of Neuroscience and Addiction Studies, School of Advanced Technologies in Medicine, Tehran University of Medical Sciences, Tehran, Iran. *; d *Department of Genetics, Faculty of Basic Sciences, Tarbiat Modarres University, Tehran, Iran. *; e *Department of Molecular Medicine, School of Advanced Technologies in Medicine, Tehran University of Medical Sciences, Tehran, Iran.*

**Keywords:** Alzheimer’s Disease, Drug Delivery System, Extracellular Vesicles, Neprilysin, Intranasal Administration

## Abstract

Alzheimer’s disease (AD) is a neurodegenerative brain disorder which has no effective treatment yet due to the blood barrier in the brain that limits the drugs with the potential of disease improvement. Extracellular vesicles (EVs) are biocompatible nanoparticles with a lipid membrane. These vesicles are secreted from various cells such as mesenchymal stem cells (MSCs) and can pass through biological barriers for transfer of information such as signals or be used as carriers for various proteins like Neprilysin (NEP). NEP is an active enzyme in the clearance of abnormal aggregated beta-amyloid sheets in the brain. In the present study, we used EVs to carry NEP for memory improvement in Alzheimer’s disease. For this purpose, bone marrow MSCs were isolated from rat femur. Stemness evaluation of established cells was characterized by differentiation potency and specific markers with flowcytometry. EVs were isolated from MSCs supernatant by ultracentrifugation and analyzed by scanning electron microscopy (SEM), dynamic light scattering (DLS) and western blotting. EVs were loaded with NEP by freeze-thaw cycle and then administrated intranasally in a rat model of the AD for 14 days. Our findings showed EV-loaded NEP caused a decrease in IL-1beta and also BAX but an increase in BCL2 expression level in the rat brain. Altogether, these data showed that EV-loaded NEP can improve brain-related behavioural function which may be mediated through the regulation of inflammation and apoptosis. These findings suggest that EV-loaded NEP can be considered as a potential drug delivery system for the improvement of AD.

## Introduction

Alzheimer’s disease (AD) is a brain degenerative disorder which is characterized by memory loss, cognitive deficiency, and dementia (1). AD will affect 100 million people by 2050 if the appropriate approaches to inhibit, slow the progression, or improve the symptoms in AD will not be specified (2). AD is known to be associated by molecular and cellular events which result in brain inflammation, and accumulation of amyloid β (Aβ) plaques and phosphorylated tau (p-tau) peptides in the hippocampus and cerebral cortex and the following restricted synaptic plasticity and neuronal signalling (3). However, based on the research, the amyloid hypothesis remains the important theory of AD pathology which has been developed alongside other concepts and targeted for drug development (4, 5). Also, reports are implied on the disrupted balance between the production and clearance of beta-amyloid in AD (6, 7). Thus, the beta-amyloid sheets are the key targeted protein in AD pathogenesis and some of the agents against amyloid sheets formation and degradation may affect disease progression (8). 

Neprilysin (NEP) also named cluster of differentiation (CD)10, is a major degrading enzyme with soluble monomeric or oligomeric (the most toxic form) forms of Aβ 1–42 and insoluble fibrils form of Aβ 1–40 in the brain (9). Various studies have indicated that inactivation or down-regulation of NEP which could be caused by ageing, is contributed to the development of AD by promoting the plaque accumulation (10). Therefore, control of the degradable (monomeric and nontoxic) form of Aβ peptide by NEP could be a preventive strategy for the clinical development of dementia (11, 12). These and other observations have resulted in considerable clinical interest in NEP protein as a novel target for AD drugs (4, 10, 13). 

Unfortunately, proteins and a large number of potent drugs have shown to be failed to cross the blood-brain barrier (BBB) (14). To overcome this problem different novel approaches have been developed (15). Recently, several reports have indicated that extracellular vesicles (EVs) could be used as a delivery system for biological drugs such as short interference RNA (siRNA) and proteins (16-18). EVs are cell-derived and small membrane vesicles with 30-1000 nm size and are secreted from various cells such as stem cells (19). Also, they could be extracted from various biological fluids such as blood, cerebrospinal Fluid (CSF), urine, and cell culture supernatant. EVs have included three main categories based on their size; exosomes (EXs, 30-100nm), microvesicles (MVs,100-1000nm), and apoptotic bodies (ABs, >1000nm) (20). These vesicles are characterized by specific markers such as CD63, CD81, CD9, and HSP70. MSCs derived EVs have low immunogenicity properties due to no or low expression of HLA-DRII (21). 

EVs are stable and biocompatible particles which are involved in cell-cell communication under both physiological and pathological conditions. These particles strongly determine their cellular origin contents (22). As a result of the mentioned functions, extracellular vesicles may consider as useful tools, for various diagnostic and therapeutic applications, specifically as a drug delivery system (23). Although it has been reported that EVs were able to target specific cell types, in some cases systemic administration has been unsuccessful. Therefore alternative routes such as in-situ and the direct intranasal administration would be effective (15, 23). Intranasal delivery is the non-invasive way for brain targeting, which also avoids drug clearance by blood circulation (24). Furthermore, slow drug absorption, overdose, and first liver metabolism are the problems which can be resolved through the intranasal route (25). A number of studies based on intranasal administration have revealed rapid delivery, high concentrations in Central Nervous System (CNS) and its effect, detected immediately after or within an hour of intranasal administration (26, 27).

In this study, EV-loaded NEP was used as a drug delivery system intranasally and disease improvement was analyzed with behavioural and histological tests in a rat model of AD. For this purpose, we purified EVs from rat bone marrow mesenchymal stem cell and characterized them with morphology, particle size, and surface markers. Isolated EVs were loaded with NEP and used in the treatment period of AD animal models. Our results proposed that intranasal delivery of EV-loaded NEP provides an effective approach for improvement in AD symptoms.

## Experimental

*Cell Culture and Verification*

Rat bone marrow mesenchymal stem cells (RBM) were extracted and established from Wistar rat femur by splashing with a syringe and transferred to the T25 flask. The cells were cultured in Dulbecco’s modified Eagle’s medium (DMEM) (Gibco, USA) which was supplemented with 2mM L-glutamine (Gibco, USA) and 10% inactivated fetal bovine serum (FBS) (Gibco, USA) in a humidified 37 °C incubator with 5% CO2. After 24 h, non-adherent cells were removed by washing with phosphate-buffered saline (PBS) (Inoclon, Iran). The cells were passaged every 2–3 days when reached about 70–80% confluence in culture flasks. 

RBM cells were characterized according to standard protocols for stem cell establishment based on two methods: marker evaluation and multi-lineage differentiation capacity (28). In brief, RBM cells were cultured on six-well plates and osteogenic induction media composed of dexamethasone 1 µM (Zahravi, Iran), ascorbic acid 200µM (Merck, Germany) and beta glycerolphosphate 10mM (Merck, Germany) was added to the cultured cells (80% confluence). Media was changed twice a week for three weeks. For adipogenic differentiation, media contained dexamethasone 1 µM (Zahravi, Iran), indomethacin 50µM (Merck, Germany), IBMX 0.5µM (Merck, Germany), and insulin 10µg/mL (Exir, Iran). Media was changed twice a week for two weeks. For staining step, the cells first were fixed for 30 min in the presence of 10% glutaraldehyde solution (Merck, Germany) in methanol (Merck, Germany) and stained in Alizarin Red (Sciencell, USA) for osteogenic differentiation in a dark place and Oil red (Biotrend, Germany) for adipocyte differentiation 60 min. All the experiments were performed in triplicate.

To markers analysis, RBM cells were detached with trypsin-EDTA0.25mM (Gibco, USA) and washed in PBS containing 2% bovine serum albumin (BSA) (Gibco, USA). Then suspended cells were stained with anti-MSC specific markers PE-conjugated (Biolegend, USA). CD34, CD45, CD90, and CD105 markers were estimated using flow-cytometry (FACScaliberTM cytometer, USA) and analyzed with FlowJo 7.6-1 software (flowjo, USA). RBM cells were used at passage numbers 3-8 for all in-vitro experiments. To evaluate the multi-lineage differentiation capacity, RBM cells were induced for 14 and 21 days in adipogenic and osteogenic conditioned medium, respectively (29).

*EVs Enrichment and Characterization*

Ultracentrifugation was used for EVs isolation which is the most common and reported techniques for vesicles isolation (30). For this, the medium of expanded RBM cells (10 × 106 cell/ T75 flask) were replaced with DMEM medium (Gibco, USA) which was supplemented with exosome free-FBS (Invitrogen, USA) in an antibiotic-free condition. Briefly, after RBM cells incubation for 24-48 h, the medium was harvested and centrifuged at 200× g for 10 min and then 2,000× g for 20 min at room temperature to remove dead cell bodies and debris and the supernatant was filtered with a 0.22μm filter unit (Sartorius, Germany). Then, the EVs were purified by ultracentrifugation (Beckman, USA) at 100,000× g for 70 min at 4 °C. The pellet was washed with 10 mL PBS and the EVs fraction protein content was assessed by the Bradford assay. Extracted EVs were suspended in PBS (500μL, 2 mg/mL total protein) and stored at −70 °C until further evaluation. Morphology, particle size, and surface proteins of EVs were characterized according to previous researches. A drop of the sample was put on a glass slide, and dried at room temperature. Scanning electron microscopy (SEM) (AMRay, USA) was used to observe the EVs morphology. The particle size of EVs was measured using a Dynamic Light Scattering (DLS) (Malvern, UK). Also, the EV markers expressions including CD9, HSP70 (Santa Cruz Biotechnology, USA) were evaluated using western blot analysis according to the published protocols for EVs confirmation (31). Briefly, 50μg of EV extracted proteins were electrophoresed and transferred to a PVDF and treated with a specific CD9, HSP70 primary antibodies overnight at 4 °C. Incubation with horseradish peroxidase-conjugated secondary antibody was performed for 90 min at room temperature. Proteins signals were detected by ECL kit using chemiluminescence reagents (Biorad, USA).

*Preparation of EVs-Loaded NEP*

EVs-loaded NEP was prepared with a slight NEP (RD system, US) mixing with EVs through the freeze-thaw cycles according to Haney protocol (32). For this purpose, EVs, isolated from conditioned media from RBM cells (10 × 106 cell/ T75, 2 mg/mL total protein), were mixed with NEP 10 μg/mL, prepared in Tris (Merck, Germany), NaCl (Merck, Germany) and ZnCl2 (Merck, Germany) solution in concentration which were recommended from the factory and incubated for 30 min, then rapidly preserved at −80 °C, and thawed at room temperature. The freeze-thaw cycle was repeated three times. To determine the efficiency of drug entrapment (also named encapsulation), the mixture was centrifuged for one hour at 100,000 g. EVs-loaded NEP was collected, washed similar to the protocol used for EV isolation in previous section and then suspended in PBS. The drug concentration in the supernatant was determined using standard curve provided with the various serial dilution of NEP concentration (0.07, 0.15, 0.3, 0.62, 1.25, 2.5, 5 ng/mL) in 280nm and UV-visible spectrophotometer (Jenway, UK). Loading efficiency (LE) is the percentage of the drug that is successfully entrapped or adsorbed into nanoparticles. It is calculated as follows: Supernatant from precipitated EVs (without loading) was used as a calibration solution for absorbance. Entrapment (or confinement) efficiency was calculated using the following formulae: (LE) = (m0 - mue /m0) ×100 which m0 is an initial total drug added and mue is free non-entrapped NEP in the supernatant (33, 34). The optimum entrapment dosage was measured based on the initial serial concentration and used for the standard curve which was mentioned before. NEP loaded EVs were aliquot and immediately stored at -70 °C. 

*Preparation of Virus Containing Mutated APP*

Production of the mutant virus was performed according to the previous study with a lentiviral vector (LV) encoding the mutant human amyloid protein precursor (APP) which bears the fAD-linked Swedish and Indiana mutations (APPSw/Ind) (35). The recombinant virus, containing mutated APP was isolated from transduced HEK-293 cell line (C10139, IBRC, Iran) supernatant and dissolved in PBS (Inoclon, Iran) to reach a titer of ~109 TU/mL. The viruses were then stored at -70 °C until the injection into the brain was done.

*Animals and Experimental Design*

Adult male Wistar albino rats (230–280g) were obtained from Neuroscience Research Center, Shahid Beheshti University (Tehran, Iran) and experiments were conducted under the internationally accepted principles for the experimental use of rats. Also, all animal studies and procedures were permitted by the Ethics Committee of Tehran University of Medical Sciences (No: IR.TUMS.REC.1394.1822). For the initial studies, the rats were housed two animal per cage under temperature and humidity-controlled conditions (25 ± 2°) in standard laboratory housing (12:12 hour light/dark cycle, with lights on at 6:00 a.m.). They were handled for two weeks before the surgery for adaptation. The rats had free access to water and food during the study. The rats were randomly divided into four groups (n = 8): (1) APP--PBS (Normal group): distilled water was injected 1μL/side in cornu ammonis (CA)1 region bilaterally; received 25µL PBS in treatment period, (2) APP+-PBS (Ctrl, control-disease group): virus containing mutated APP was injected 5μL/side in CA1 regions bilaterally; received 25µL PBS in treatment period,(3) APP+-EVs (EVs, treatment control group): virus containing mutated APP was injected 5 μL/side in CA1 regions bilaterally; received 25 µL EVs in treatment period, (4) APP+-EV-loaded NEP (EV& NEP, treatment group) virus containing mutated APP was injected 5 μL/side in CA1 regions bilaterally; received 25 µL EV-loaded NEP in treatment period. All injections were performed intranasally for 2 weeks, 43 days after surgery.

*Surgery*

The rats were sited in a stereotaxic tool (Stoelting, USA) after anesthetization with an intraperitoneal injection of combined ketamine (100 mg/kg) and xylazine (10 mg/kg) (Alfasan, Netherlands). The stereotaxic device was set up for dorsal hippocampus and the surgery was performed according to the atlas of the rat brain (36, 37). The solution containing viruses (5μL/side) was injected bilaterally into the CA1 region of the hippocampus using a 5μL Hamilton syringe (Hamilton, Reno, Nevada). The microinjections were performed in 15 min, and the needles were left in place for 5 min to help the distribution of the injected viruses. The mutated viruses accumulate intra-neuronal Aβ in the brain hippocampus.

*Morris Water Maze test*

Morris water maze (MWM) test was evaluated through the acquisition phase as spatial learning and probe trial as memory evaluation. The apparatus consisted of the maze tank surrounded by pictures on the walls and dark circular water-filled pool and a platform put in the one quadrant of the maze and 2 cm below the water surface (38, 39). The platform was positioned in the same quadrant on every trial and provides only the escape from the water. The animal movement in the tank was recorded with a video tracking system (Panasonic Inc., Japan) placed properly at the top of the maze apparatus. EthoVision version XT7.0 (Noldus, Netherlands), a video tracking software was used for investigation of behavioural experiments automatically through the 60s. The training began on day 50th after stereotaxic injection of the above-mentioned experimental groups (7 days after treatment). Four days (50th-53th after virus injection) were included four training trials per day (the 60s at 90s intervals) and the day after the final training period, a single trial probe was performed in the pool without the platform. All tests were accomplished between 9am to 15 pm.

*Passive Avoidance test*

Passive avoidance test was used to analysis of learning and memory maintenance abilities, in control, EVs and EV-loaded NEP administered animals. In this test, the rats were allowed to habituate to the trial room at the training day, one hour before the experiment and then were placed in the shuttle box composed of a light and a dark partition joining each other through a gate. Then, each rat was placed in the light part, back to the closed gate which opened after 10s. When a rat entered the dark part so that all four limbs were on the dark side, the gate was closed, and a foot shock (1mA, 1.5s) was provided. The rats were then placed in the light part and another foot shock was applied if reentered to the dark side during 120s. During the test phase, the rat latency to enter the dark compartment (step-through latency; STL) and the total time spent in the dark part was recorded. All experiments were done between 9am and 15pm (40).

*Y-maze test*

Y-maze spontaneous alternation is a well-known behaviuoral test for measuring working memory during a single period in a symmetrical Y-maze device composed of three arms and have been described previously (41, 42). The test is based on the rats’ preference for investigation of the arm which was not visited at the two previous selections. Each experimental rat was placed in the centre of the Y-maze and allowed to free access to all arms and move without restriction during an 8-min period. The arm entry was recorded when the rat hind limb was completely in the arm. Then, the sequence and the total number of arms entered were monitored and recorded. Alternation was defined as totally entries into all arms so that there was no repetition in a triple set. Percentage of alternation is calculated as the following formula: ((number of alternation)/ (total number of arm entries − 2)) × 100.

*Tissue Preparation and Immunohistochemistry*

Immediately after the behavioural testing achievement, each group consisting of 8 rats (Normal, Ctrl, EVs, EV& NEP) were euthanized using CO2 inhalation, and the brains were removed and cut in half sagittal. One hemisphere of each brain (Randomly) was immediately cryopreserved in liquid nitrogen and stored at −80 °C for Real-Time PCR, and the other was immersed in 4% paraformaldehyde (Merck, Germany) in 0.1M PBS (Inoclon, Iran) overnight and used for histological evaluates. After de-paraffinization, the tissues were dissected and post-fixed with 4% paraformaldehyde in PBS. The coronal sections with the thickness of 7μm were prepared and stained with 0.1% cresyl violet acetate solution (Sigma, USA), a neurological stain. For the morphological study, three histological sections of the CA1 region were used (200x magnifications). 

For analysis of Aβ plaques, 16m-thick sections were blocked with 5% BSA (Sigma, USA). Briefly, cell nuclei were stained with DAPI and the samples were immune-stained with Anti-Aβ monoclonal antibody (abcam, USA) for 16 h at 4  °C and a secondary antibody, anti-rat IgG conjugated to HRP (abcam, USA) for 30 min at RT. Images were achieved using confocal microscopy (Olympus, Japan). The Aβ plaques were considered as the percentage of the immune-positive zone (positive pixel) to the investigated zone (total pixel). The results were quantified using ImageJ software (National Institute of Health, USA).

*RNA extraction and Real-time quantitative PCR*

The hippocampus region was isolated from the rat brain and transferred into sterile cryovials and immediately stored in −80 °C. RNA extraction was conducted using the RNeasy Plus Mini kit, (Qiagen, USA) and the protocol provided by the manufacturer. RNA quantity was evaluated by NanoDot spectrophotometer (Hercuvan, Malaysia). Also, agarose 1% (Sigma, USA) gel electrophoresis was used to assess samples quality and integrity.

Real-time quantitative PCR (qPCR) was performed by the Prime script RT kit (TAKARA, Japan) and 1μg of RNA and using Step one plus ABI system (Applied Biosystems, USA). Glyceraldehyde-6-Phosphate Dehydrogenase (GAPDH) was used as a reference gene. qPCR was performed under the following conditions: The PCR system included 10µL SYBR EX Taq-Mix, 0.5µL Forward and 0.5µL Reverse primers (Table1), 1µL cDNA and 8µL ddH2O in 20µL final volume. Each sample was experienced in triplicate reactions. PCR conditions were according to the procedure: initial denaturation at 95 °C for 10 min; 40 cycles of 95 °C for 1 min, 60 °C for 40 sec, 72 °C for 30 sec, and 72 °C for 1 min. Relative expression of mRNA was calculated using 2−ΔΔCq method.

*Statistical analysis*

All data were represented as mean ± SD on curves and processed using GraphPad prism6 software (La Jolla, USA). Two-way ANOVA followed by Tukey’s test was used to determine the difference between the groups. Statistical significances are set at <0.05, p < 0.01 and p < 0.0001.

## Results

*RBM cells isolation and characterization*

**Figure 1:**
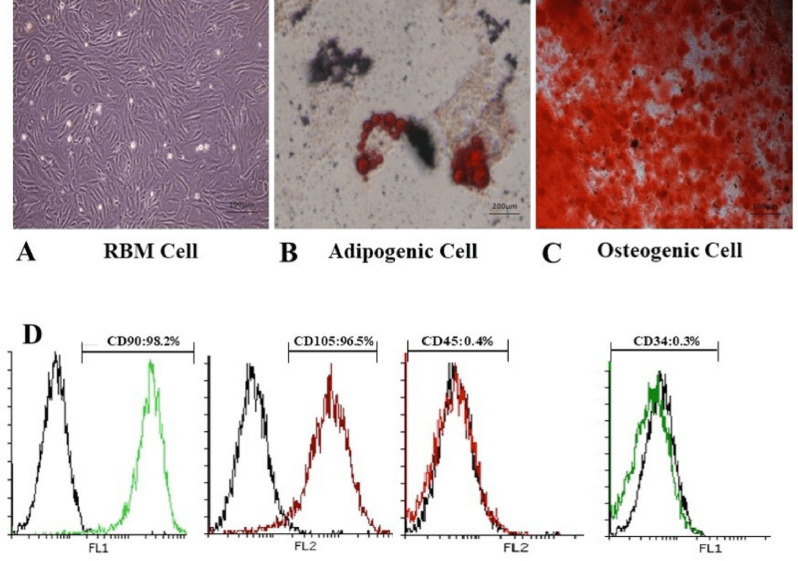
Characterization of rat bone marrow cells. Differentiation of RBM cells in induction media for osteogenic and adipogenic differentiation. (A) Established RBM cells after 2 passages were used as control for confirmation of differentiation. (B) Differentiation to adipogenic lineage was confirmed with lipid condensations through cell cytoplasm stained by Oil red, 14 days after culture in induction medium compared to RBM cells. (C) Calcified structures were evaluated in osteocyte stained by Alizarin red, 21 days after culture in induction medium. (D) Evaluation of RBM cell markers expression showed more than 95% in CD90, CD105 and less than 0.5% in CD45 and CD34 markers by flowcytometry which verified stem cell property of RBM cells

RBM cells were isolated from rat femurs and cultured in DMEM supplemented medium. The MSC-like population is adherent and monolayer which helps to effective extraction. For this purpose, harvested cells were cultured in tissue treated culture dishes; thus, only MSCs adherend and retained in culture. After 7-10 days, the majority of non-adherent cells were removed during the medium exchanges. The remaining cells had a heterogeneous fibroblastic-like appearance and displayed separate colony formation. The cells were further passaged to achieve a homogenous population with spindle-shaped morphology (Figure. 1A). These cells should be passaged at least eight times to prevent the differentiation of the RBM cells. 

MSCs are defined as multipotent stem cells which can be able to differentiate to osteoblastic and adipocytic lineages. Osteogenic differentiation assay was evaluated by induction media to confirm the multipotency of the RBM cells. After three weeks, the cells widely differentiated into osteoblasts and positive alizarin staining was observed after three weeks in samples. Adipogenic differentiation assay confirmed the lipid drops accumulation in cell cytoplasm stained by oil red after two weeks and identify lipid-laden fat cells (Figure. 1B). Also, Osteocytes were embedded with heavy mineralization and calcium oxalates deposition in the extracellular matrix (Figure 1C). These results were not observed in the undifferentiated MSCs.

Generally, a standard for the identification of MSC is the positive presence of CD73, CD90, and CD105 in cell surface while being negative for CD34, CD45. For RBM cells characterization, the cells were evaluated with marker analysis through flowcytometry. The results showed that RBM cells express CD90, CD105 as positive markers more than 95% and are negative for CD34 and CD45 markers less than 0.5% (Figure 1D).

*EVs isolation and loading with NEP*

**Figure 2:**
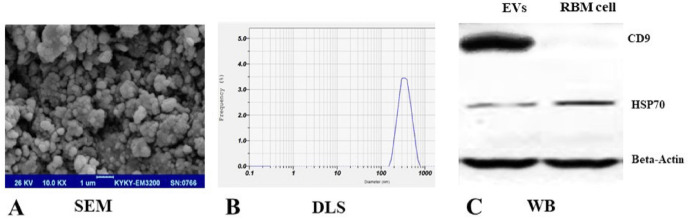
Characterization of RBM cells-derived EVs**. **(A) Scanning electron microscopy (SEM) showed spheroids but slight heterogenicity in EVs population. (B) Size distribution of RBM cells-derived EVs were estimated using DLS and showed an average 223.43 ± 18.48nm size. (C) Western blot investigation was used and confirmed protein markers expression, CD9 and HSP70 in EVs derived from RBM cells. Beta-actin was used as control protein

Since FBS supplement in cell culture supernatant would affect results, the medium was changed with exosome depleted serum and EV particles isolated after 48-hour incubation from RBM cells supernatant by use of ultracentrifugation. RBM derived EVs were purified according to the protocols described previously (30). SEM indicated the EVs population with the spheroid morphology but light heterogeneity in size (100-300nm) (Figure 2A). Analysis of DLS showed that the mean size of EVs was 223.43 ± 18.48nm and a polydispersity index (PDI) of 0.36 ± 0.05 (Figure 2B). 

Zeta potential of a freshly EV solution was -32mV which helps EVs stability. Zeta potential in EVs which were loaded by NEP was cationic, +28mv. Also, results from western blot analysis verified the EV markers expression; CD9 and HSP70 in RBM derived EVs (Figure 2C). Loading efficiency was analyzed to use the optimal dose of entrapped NEP into EVs in relative to initial drug concentration. The experiment revealed that 0.625 ng/mL NEP has the highest LE (30 ± 1.7%) compared to lower NEP dosage (18.5-26.5 ± 1.3%) for entrapment. All experiments were performed in triplicate assays and significant changes in entrapment efficacy were not seen in higher doses of NEP quantity performance.

*Memory improvement induced with EV-loaded NEP administration*

**Figure 3:**
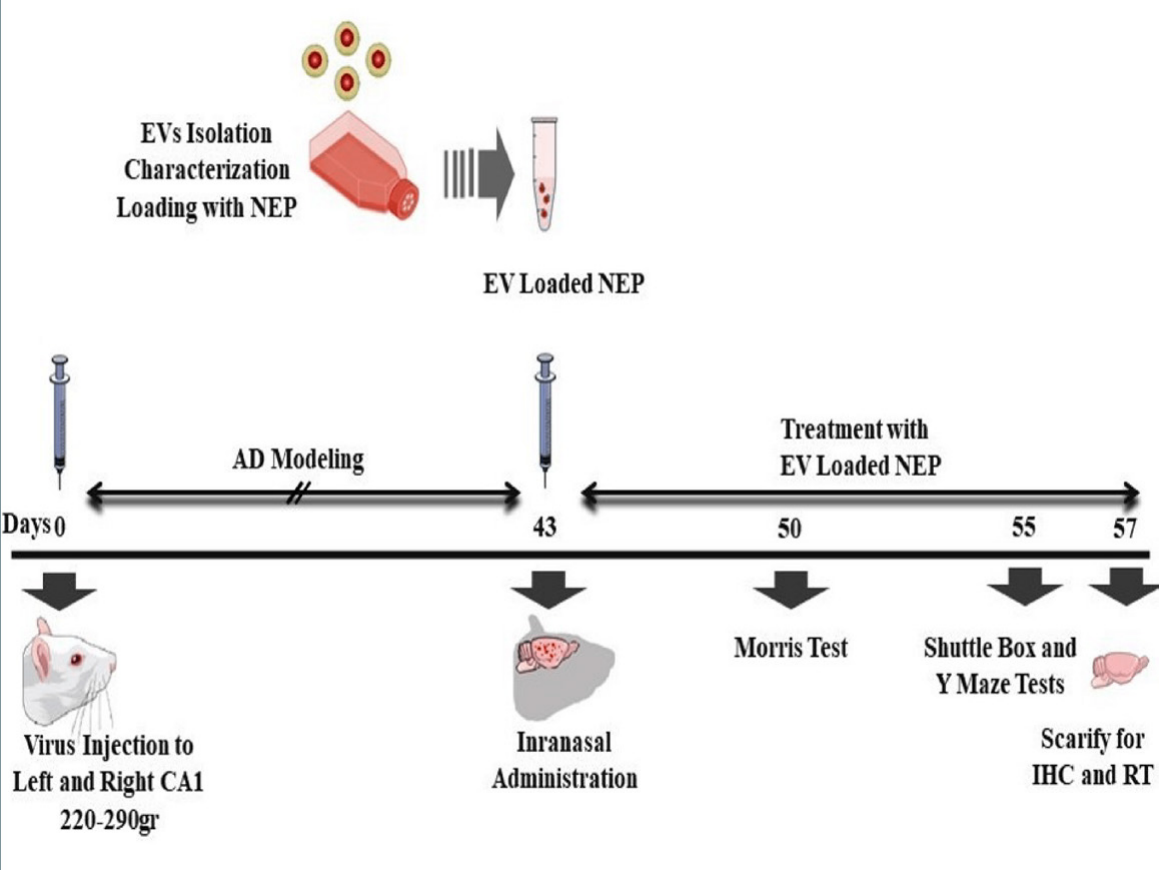
Schematic diagram of the experimental protocol. Animals received intra-CA1 injection of virus (5μg/side) bilaterally. Animals treated by PBS in control, EVs and EV-loaded NEP at 43th day intranasally. Spatial learning and memory were measured 7 days after drug administration. Working memory and passive avoidance memory were evaluated via Y-maze test and shuttle box respectively at 12th day after treatment. Animals were sacrificed on 14th day after treatment and the hippocampus tissues were harvested for further assessments

Effects of consequence administration of EV-loaded NEP on memory were measured with Morris water maze at 7th days, passive avoidance at 12th days and Y-maze at 14th days after NEP treatment. The used process in our experiment has been schematically summarized in Figure 3. 

**Figure 4:**
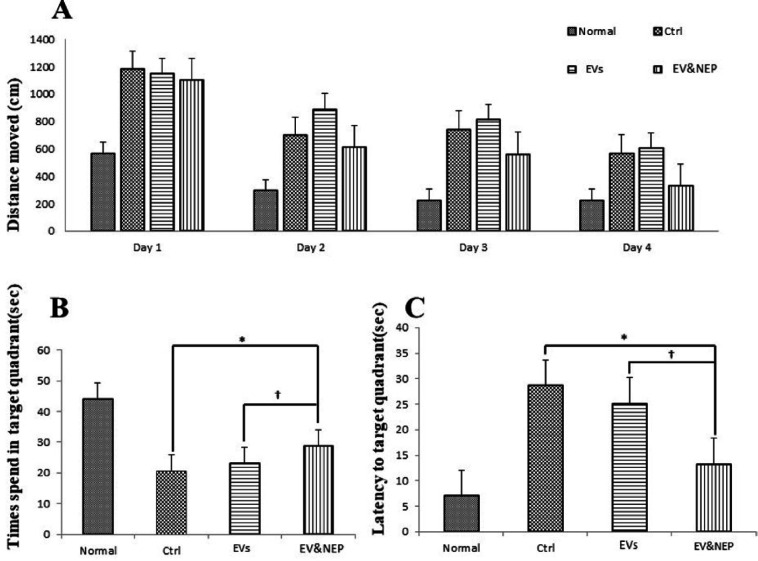
The effect of intranasal EV-loaded NEP administration on spatial memory impairment. (A) Distance moved with the platform during four training days, (B) time spent in target quadrant and (C) latency to target quadrant and with the removed platform in the probe trial day. MWM test showed a significant difference in animals which treated by EV-loaded NEP compared to the control group (*p *< 0.01), and EVs-treated animals (*p *< 0.05); (N = 8 in each group; the differences between groups were determined by ANOVA followed by Tukey test. † *p *< 0.05, ∗*p *< 0.01). Defined groups are Normal, Ctrl: received PBS, EVs: received EVs, EV&NEP: received EV-loaded NEP in the treatment period

In the MWM test, rat disease model which received EV-loaded NEP in the treatment period, spent significantly shorter searching time for the hidden platform and thus decrease in distance movement compared with the EVs and the control groups during four days training (Figure 4A, p<0.01). Also, EV-Loaded NEP group spend more time in the target quadrant (with the hidden platform) than in other quadrants in comparison to the EVs and control groups (Figure 4B, p<0.01). Furthermore, EV and EV-loaded NEP-treated animal groups showed a considerable reduction in escape latencies over consecutive training trials but was more significant in EV-loaded NEP-treated animal than EVs group (Figure 4C, p < 0.01).

**Figure 5:**
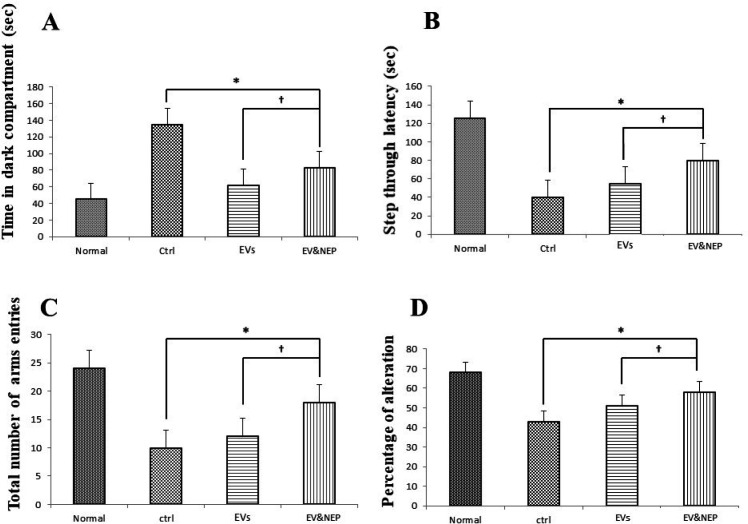
The Effects of intranasal EV-loaded NEP administration on passive avoidance shuttle box and working memory performance on Y-maze. (A) Time in dark compartment, B) step thorough latency on shuttle box, EV-loaded NEP enhanced memory retention compared to EVs (*p*<0.05) and control groups (*p *< 0.01). (C) total arms entries and (D) percentage of alteration in the Y-maze test, significant difference was observed between EV-loaded NEP compared to the EVs (*p *< 0.05) and control groups (*p *< 0.01); (N = 8 in each group, the differences between groups were determined by ANOVA followed by Tukey test. †*p *< 0.05, ∗*p *< 0.01). Defined groups are Normal, Ctrl: received PBS, EVs: received EVs, EV&NEP: received EV-loaded NEP in the treatment period

To survey another hippocampus-dependent memory, the passive avoidance test was performed in which the animals learn to avoid electric shock. Two-way ANOVA analysis showed that EV-loaded NEP treated animals indicated lower latency entrance into the dark part in contrast to the EVs (p < 0.05) and control groups (p < 0.01) (Figure 5A). In addition, step through latency was significantly increased in EV-loaded NEP group compare to EVs (p < 0.05) and control groups (p < 0.01) (Figure 5B). This parameter had no significant changes in EVs treatment group in comparison with the control group. 

In Y-maze test, two-way ANOVA analysis showed that EV-loaded NEP-treated animals indicated a significant increase in the total number of arms entries (Figure 5C) and alternation behavior tests in comparison to the EVs (p<0.05) and control group (p < 0.001), indicating memory improvement, while no significant difference was detected in EV administration compared with the control group (Figure 5D).

*EV-loaded NEP decreased neuronal damage and Aβ plaques*

**Figure 6:**
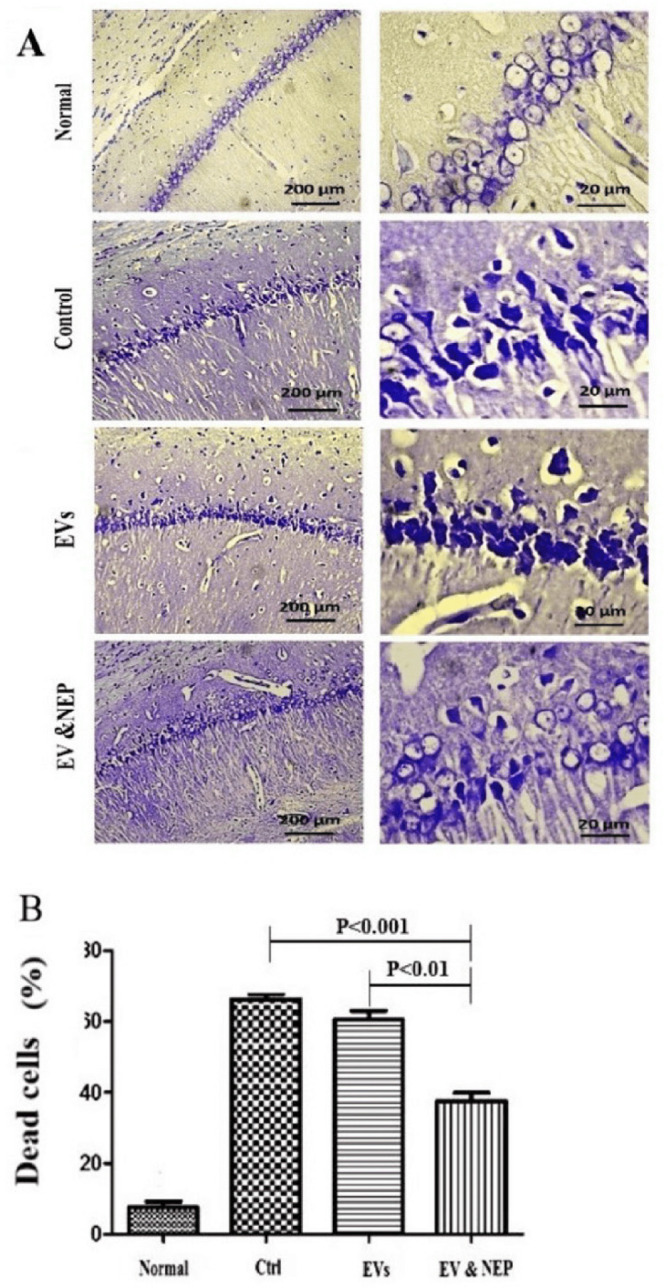
The effect of intranasal EV-loaded NEP administration on hippocampus neuronal damage. (A) Representative micrographs of Nissl stained sections resulted in Normal, Control, EVs, and EV-loaded NEP groups, Scale bars: 200μm and 20μm and (B) percentage of dead cells, indicated significantly decrement in CA1 neuronal cell death count in EV-loaded NEP delivered rat brains compared to control (*p *< 0.001) and EVs (*p *< 0.01) groups after 14 days treatment; (n = 4 in each group, the dead cell results were quantified with ImageJ software and the differences between groups were determined by ANOVA followed by Tukey test). Defined groups are Normal, Ctrl: received PBS, EVs: received EVs, EV&NEP: received EV-loaded NEP in the treatment period

Cresyl violet or Nissl staining was performed to evaluate neuronal lost. Intranasal administration of EV-loaded NEP can remarkably reduce histological changes and neural damages which would be a result of beta-amyloid degradation in comparison to EVs (p < 0.01) and the control groups (p < 0.001) (Figure 6). 

**Figure 7:**
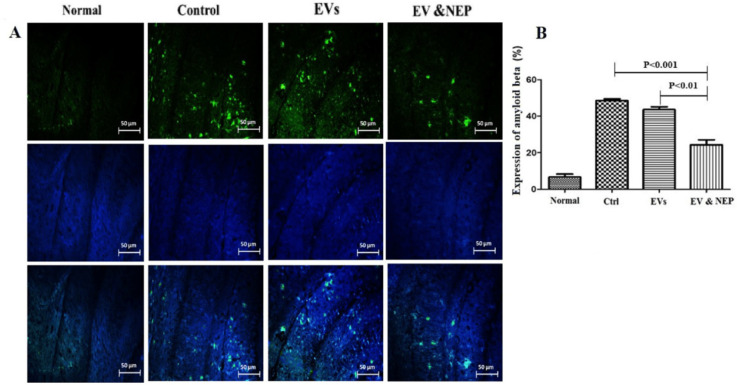
The effect of intranasal EV-loaded NEP administration on beta-amyloid plaques accumulation**. **(A) Representative micrographs of immunofluorescence staining of beta-amyloid proteins in Normal, Control, EVs, and EV-loaded NEP groups, Scale bar: 50μm. Cell nuclei were counterstained with DAPI and (B) Expression percentage of Aβ plaques, significantly decreased in EV-loaded NEP group was observed in comparison to control (*p *< 0.001) and EVs (*p *< 0.01) groups after 14 days treatment. Images were represented from at least 3 sections per animal in each experimental group after 14 days treatment; (n = 4 in each group, the results were quantified with ImageJ software and the differences between groups were determined by ANOVA followed by Tukey test). Defined groups are Normal, Ctrl: received PBS, EVs: received EVs, EV&NEP: received EV-loaded NEP in the treatment period

**Figure 8:**
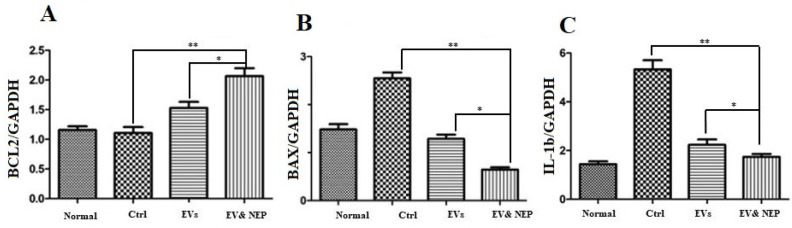
The effect of intranasal EV-loaded NEP administration on inflammation suppression and apoptotic processes at mRNA level in brain. Real-Time analysis of (A) BCL-2 as an anti-apoptotic factor, (B) BAX as a pro-apoptotic and (C) IL-1 beta as an inflammation factor in the brain hippocampus of the Normal, Control, EVs, and EV-loaded NEP groups. Down regulation of BAX and IL-1 and upregulation of BCL-2 were observed in EV-loaded NEP group in comparison EVs (*p *< 0.01) and control groups (*p *< 0.0001); (n = 4 in each group, the differences between groups were determined by ANOVA followed by Tukey test, ∗∗*p *< 0.0001, ∗*p *< 0.01). GAPDH gene was used as control gene. Defined groups are Normal, Ctrl: received PBS, EVs: received EVs, EV&NEP: received EV-loaded NEP in the treatment period

Also, beta-amyloid aggregation is one of the major hallmarks of AD which is indicated by specific immunostaining. The results revealed the reduced level of Aβ in the hippocampus of the rats through EV-loaded NEP treatment during 14 days, compared to EVs (p < 0.01) and control groups (p < 0.001) (Figure. 7A and 8B). There was no significant difference between EVs and the control groups in Nissl staining.

*Intranasal administration of EV-loaded NEP inhibited inflammation and apoptosis*

Interleukin-1(IL-1) beta is a pro-inflammatory cytokine which leads to neuroinflammation, cell death and neurodegeneration in the brain. To determine whether intranasal delivery of the EV-loaded NEP could prevent inflammation and the following pathology as apoptosis, IL-1beta and BCL2-associated X protein (BAX) and B-cell lymphoma 2 (BCL2) family proteins which play a critical role in tuning cell death processes in the brain were assessed at mRNA level. All curves were linear in the range (R2>0.999) and invalid data were not counted in the final analysis. 

The results from the experiments showed a statistically significant down-regulation expression level in the pro-apoptotic gene (BAX) but up-regulation in an anti-apoptotic gene (BCL-2) when compared to the control (p < 0.0001) and EVs (p < 0.01) groups. Collectively, these data support that EV-loaded NEP inhibits inflammation and apoptosis (Figure. 8A and 8B). Also, IL-1beta decreased significantly in EV-loaded NEP group incomparison to the group which received EVs (p < 0.01) and the disease control group (ctrl) (p < 0.0001). There was no significant difference between EVs and the control groups in Aβ plaques specific immunostaining (Figure. 8C).

In this research study, our results showed that EVs which were loaded with NEP has a significant effect on the improvement of spatial learning and memory deficits with the decrease in the number of beta-amyloid plaque in the hippocampus of the adult rat brain. 

In the present research, we established RBM cells from rat bone marrow according to standard protocols for mesenchymal stem cell isolation and confirmation. The cells had adherent Fibroblast-like morphology, immunophenotyping based on positive CD90 and CD105 and negative CD34 and CD45 markers and multilineage differentiation potency which were introduced by the international society for cell therapy (ISCT) guidelines (43). Experiments on MSCs characterization confirmed the potential of differentiation to osteogenic and adipogenic lineages in an appropriate induction medium which showed consistency with other establishment-based studied for characterization of MSCs (44). 

Furthermore, it is known that MSCs can produce and secrete a great variety of EVs, therefore we isolated and characterized EVs, based on methodology presented by international society for extracellular vesicles (ISEV) (45, 46). SEM showed isolated EVs with round-shape morphology and marker analysis expressed two standard available markers (CD9 and HSP70) (47). Although, EVs size was not studied as a targeted variable in our research, DLS measurement indicated that the average size of purified EVs was 223.43 ± 18.48 nm and variation in size (100-300nm) was observed which could be related to the use of ultracentrifugation method in our study compared with column based purification methods which usually results in homogenized vesicles. Regarding that EVs with Zeta potential greater than +25mV or less than -25mV usually show long-term stability and prevention of aggregation, our experiment revealed -32mV Zeta potential which leads to particle stability (48). Additionally, transportation of nanoparticles such as EVs comprises from electrostatic interaction between a delivery system and targeted cell membrane which contains negatively charged sialic acid residue on the luminal surface of the brain capillaries. Nanoparticles with positive zeta potential can effectively transport from BBB (49). In this regard, we used NEP in Tris buffer which contains positive amine groups and can convert anionic EV to cationic ones which could facilitate transport and absorption of the particles across the BBB. Our results confirmed other research outcomes in the delivery of positive proteins and nanoparticles to the brain (50).

Moreover, various studies showed EVs could be considered as a delivery system for hydrophobic and hydrophilic drugs such as curcumin and anticancer antibodies, and also smaller EVs (about 100-300nm such as our isolated EVs) could be helpful for absorbance and passing through BBB. Our results are in accordance with other studies which confirmed the role of EVs in promoting drug delivery to the brain (51-53). In the process of EV loading, the highest level of entrapment efficacy was 30% with initial drug concentration ≥0.625ng/mL and there were no significant changes in higher drug dosage. 

Recently several studies have focused on the reduction of beta-amyloid by drugs or biological factors and showed EVs derived from MSC potentially contains activated NEP enzyme as anti-aggregation agent but it seems necessary to evaluate the function of recombinant NEP protein in EV-loaded NEP in comparison to the EVs with naturally NEP on beta-amyloid sheets (56-58). Our result confirmed the EVs positive effect in the clearance of beta-amyloid sheets which could be related to wild-type NEP in MSCs-derived EVs but this effect remarkably increased in EV-loaded NEP. This could be a shred of evidence that there is no conflict between the function of recombinant NEP and wild type NEP. 

Also, IHC results from EV-loaded NEP strongly indicated the reduction of Aβ plaques in the hippocampus of the rat brain. This was followed by reduced inflammation through suppression of IL-1beta production which can lead to neural death factors. 

Although some studies are based on NEP treatment using viruses mediated expression, there are several reports on virus-based methods disadvantages due to oncogenes activation (59). Furthermore, molecular regulation of the drug dose is an important factor which should not be neglected and is not easily controllable in-vivo in virus-mediated therapies. In addition, APP transgenic mice with the NEP protein overexpression, have exhibited improved degradation of Aβ but no improvement in memory retrieval (54). Therefore, using EVs through intranasal route provides a non-invasive and efficient virus-free approach for NEP delivery into the CNS at determinable doses and restricted side effects on peripheral organs.

Several studies have focused on the biocompatible vehicle such as EVs for delivery systems in the cell-free therapy of diseases, they mainly emphasized on EVs protection of the cargo from degradation and unwanted changes (32). On the other hand, drug delivery using EVs has solved the problem with blood brain barrier transportation. Recent progress in drug delivery has indicated that intranasal rout is a non-invasive drug administration. This approach might lead to escapes from the systemic circulation clearance and direct access to the brain by going through intra- and extra neuronal pathways which were further proved by our findings and IHC results indicated that intranasal administration of EV-loaded NEP reduced beta-amyloid plaques in the hippocampus. Also, our results showed that treatment with EV-loaded NEP meaningfully improved the cognitive behavioral including spatial memory and learning function tests in APPSw/Ind rat compared with EVs or PBS treated animals 

Previous studies using EVs derived from mesenchymal stem cells demonstrated the effective roles of EVs in immunomodulation and suppression of oxidative stress in various brain diseases such as AD (55, 56).Our molecular analysis showed that the results are consistent with the experiments which have reported impairment in behavioural test in addition to reduction of inflammation and Aβ aggregation. This effect was significantly better in EVs-loaded NEP in comparison to EV and control groups and was followed by suppression of apoptosis. This event was indicated through up-regulation of BCL-2 and down-regulation of BAX which finally led to inhibition of neuronal cell death in the hippocampus. These findings propose the potential of EVs as an efficient drug delivery system to the brain (57). In summary, the results from our research suggest that EVs-loaded NEP have a positive effect on cognitive performance improvement in AD-like neurodegenerative context and could be used as a protein delivery system through intranasal route. 

**Table1:** Primer sequences used for Real-Time PCR

**Gene expression**	**Sequence**	**Reference **
BCL2-associated X protein (BAX)	F: CTGGATCCAAGACCAGGGTG R: GTGAGGACTCCAGCCACAAA	(58)
Interleukin 1 beta (IL-1b)	F: TGCCACCTTTTGACAGTGATG R: TGATGTGCTGCTGCGAGATT	(59)
B cell leukemia/lymphoma 2 (BCL2)	F: GCGTCAACAGGGAGATGTCA R: GCATGCTGGGGCCATATAGT	(60)
Glyceraldehyde-3-phosphate dehydrogenase (GAPDH)	F: CCCTTAAGAGGGATGCTGCC R: TACGGCCAAATCCGTTCACA	(60)
